# Turnover Intention Among Nurses and Midwives in Obstetrics Departments: A Nationwide Cross-Sectional Survey in Japan

**DOI:** 10.7759/cureus.117162

**Published:** 2026-09-29

**Authors:** Masatoshi Ishikawa, Ryoma Seto, Michiko Oguro, Yoshino Sato, Mayo Ogawa, Izumi Katagiri, Mini Kaneko

**Affiliations:** 1 Research Institute, Tokyo Healthcare University, Tokyo, JPN

**Keywords:** japan, midwife, nurse, obstetrics, turnover intention, working hours

## Abstract

Objective

Nurse turnover is a global challenge, and working conditions have been identified as a major contributing factor. Obstetrics departments, in particular, are characterized by high workload intensity due to deliveries and emergency care; however, nationwide evidence on turnover intention among nurses and midwives working in this setting remains limited. This study aimed to examine the correlates of turnover intention among nurses and midwives working in obstetrics departments in Japan and to identify associated individual and organizational factors.

Methods

A nationwide web-based questionnaire survey was conducted among nurses and midwives working in obstetrics departments at 1,170 hospitals in Japan. Turnover intention was assessed using two items: intention to leave the current hospital and intention to leave the nursing or midwifery profession, both measured by frequency. Multivariate logistic regression analyses were performed, with high-frequency turnover intention (at least once per week) as the dependent variable and respondents’ demographic characteristics and working conditions as independent variables.

Results

Valid responses were obtained from 2,043 nurses and midwives at 474 hospitals, corresponding to a hospital-level response rate of 40.5%. Overall, 494 (24.2%) respondents reported thinking about leaving their current hospital at least once per week, and 199 (22.2%) reported thinking about leaving the nursing or midwifery profession at least once per week. Longer weekly working hours were associated with greater intention to leave the current hospital, with a dose-response pattern across the 50-59, 60-69, and ≥70-hour categories. Holding a head nurse position, working in facilities with more midwives or at least 10 advanced midwives, and employment at university hospitals were associated with lower intention to leave the current hospital. Intention to leave the profession was higher among respondents working ≥70 hours per week, whereas holding a head nurse position, having at least 10 advanced midwives, employment at university hospitals, and working outside urban areas were associated with lower intention to leave the profession.

Conclusions

Among nurses and midwives working in obstetrics departments in Japan, very long working hours were associated with higher turnover intention, whereas organizational factors-including managerial roles, staffing resources within the same profession, university-hospital employment, and regional setting-were associated with lower turnover intention. Based on nationwide, department-specific data, this study highlights the importance of addressing turnover intention not only as an individual issue but also as an organizational and structural problem, providing evidence to inform workforce retention strategies and policy discussions aimed at improving working conditions.

## Introduction

Shortages of nurses and midwives represent a serious challenge to healthcare systems worldwide, exerting substantial impacts on the quality and safety of care as well as on the sustainability of healthcare delivery systems. International studies have repeatedly demonstrated that high nurse turnover rates are associated with increased patient mortality, adverse events, burnout among healthcare professionals, and reduced healthcare system performance [1-3]. Accordingly, nurse turnover is now regarded not merely as a personnel issue, but as a structural challenge affecting healthcare systems globally [4]. In this study, turnover intention was defined as the self-reported frequency of thoughts about leaving either the current hospital or the nursing/midwifery profession.

Previous research has shown that turnover intention among nurses is strongly associated with working conditions and organizational factors, including long working hours, excessive workload, inadequate staffing levels, insufficient workplace support, and reduced professional autonomy [5-8]. Systematic reviews and meta-analyses have further demonstrated that excessive workload and long working hours adversely affect both the physical and mental health of nurses, thereby increasing burnout and workforce attrition [9,10]. Recent international studies have also reported increasing burnout, psychological distress, and turnover intention among nurses during periods of heightened clinical demand [11]. In addition, managerial support, educational opportunities, and appropriate staffing structures have been reported to function as protective organizational factors that improve retention and job satisfaction among nurses [12-14].

Theoretical frameworks of turnover intention suggest that turnover intention is influenced not only by individual burden but also by organizational and environmental factors, including workload, workplace support, staffing resources, and professional autonomy [15].

In addition, the Theory of Planned Behavior proposes that behavioral intentions are shaped by attitudes, perceived behavioral control, and social or organizational contexts [16]. Similar theoretical approaches have recently been applied in occupational health research examining turnover intention and behavioral outcomes [17]. Therefore, excessive workload and unpredictable work demands may increase psychological distress and burnout, whereas supportive organizational structures and adequate staffing may function as protective factors that improve retention.

Obstetrics departments constitute a clinical setting characterized by unpredictable and high-intensity workloads, including deliveries, emergency cesarean sections, maternal and fetal emergencies, and continuous perinatal monitoring. In addition to physical workload, obstetrics professionals may experience substantial psychological burden related to responsibility for both maternal and neonatal outcomes. These characteristics may contribute to burnout, reduced job satisfaction, and turnover intention among nurses and midwives. Because obstetric care relies heavily on specialized professionals such as midwives, staffing structure and skill mix may play particularly important roles in workforce retention within obstetric departments.

In Japan, chronic shortages of nursing staff remain a major healthcare policy issue. Although work-style reform policies for physicians have been promoted institutionally in recent years, comparatively limited attention has been paid to the working conditions and retention of nurses and midwives, despite increasing workload demands in obstetric care settings. Surveys conducted by the Japanese Nursing Association have repeatedly identified harsh working conditions as a major reason for nurse turnover [18]. However, nationwide evidence specifically focusing on turnover intention among nurses and midwives working in obstetrics departments remains limited.

Therefore, this study aimed to examine the correlates of turnover intention among nurses and midwives working in obstetrics departments in Japan, with particular attention to organizational factors, working conditions, and workforce support.

## Materials and methods

Participants and recruitment

A total of 1,170 hospitals that publicly reported obstetrics services under the Japanese Bed Function Reporting System [19] were identified as the study population. A nationwide web-based questionnaire survey was conducted targeting nurses and midwives working in obstetrics departments at hospitals throughout Japan. Hospitals without obstetrics departments were excluded. Responses with missing data regarding turnover intention variables were excluded from the multivariate analyses. The final sample size was considered sufficient for logistic regression analyses based on the recommended events-per-variable approach.

Data collection

On September 1, 2024, a survey request was sent to the nurse managers responsible for nursing staff in the obstetrics departments of these hospitals. A web-based questionnaire survey was conducted using a self-administered questionnaire method. The survey period ended on September 30, 2024. Web-based questionnaires were distributed to eligible hospitals nationwide.

To improve response quality, incomplete questionnaires and responses with missing turnover intention data were excluded from multivariate analyses. Responses were reviewed for completeness and consistency of response patterns before inclusion in the final dataset.

Cases with missing data for turnover intention variables were excluded from multivariate analyses. Missing data for other variables were treated as missing values and were not imputed.

Measurements

First, respondents’ characteristics were described, including age, professional qualifications, job position, highest educational attainment, weekly working hours, number of full-time physicians, number of full-time midwives, number of advanced midwives, annual number of deliveries, ownership type of the employing institution, and regional characteristics (Table 1).

**Table 1:** Characteristics of respondents

No. of Respondents	2,043	
Gender		
Female	2,041	99.9%
Male	2	0.1%
Age		
<30	434	21.2%
30-39	503	24.6%
40-49	602	29.5%
50-59	443	21.7%
≥60	61	3.0%
Professional qualification		
Midwife	1,640	80.3%
Registered nurse	1,463	71.6%
Licensed practice nurse	99	4.8%
Advanced midwife	397	19.4%
Job position		
Staff nurse	1462	71.6%
Charge nurse	360	17.6%
Head nurse	180	8.8%
Other	41	2.0%
Highest educational attainment		
Vocational school	989	48.4%
Junior college	353	17.3%
University	488	23.9%
Graduate school	146	7.1%
Other	67	3.3%
Working Hours per Week		
<40 hours	463	22.7%
40-49 hours	1263	61.8%
50-59 hours	244	11.9%
60-69 hours	51	2.5%
≥70 hours	22	1.1%
Number of full-time physicians		
<5	841	41.2%
5-9	858	42.0%
≥10	293	14.3%
Unknown	51	2.5%
Number of full-time midwives		
<10	389	19.0%
10-19	763	37.3%
20-29	450	22.0%
≥30	384	18.8%
Unknown	57	2.8%
Number of advanced midwives		
<5	803	39.3%
5-9	564	27.6%
≥10	222	10.9%
Unknown	454	22.2%
Annual number of deliveries		
None	45	2.2%
<200	509	24.9%
200-400	714	34.9%
≥400	642	31.4%
Unknown	133	6.5%
Foundational Entity of Employer		
Public	1,262	61.8%
Private	551	27.0%
Public University	131	6.4%
Private University	99	4.8%
Employer’s Regional Classification		
Urban	693	33.9%
Intermediate	1,062	52.0%
Rural	288	14.1%

Age was categorized into five groups: under 30 years, 30-39 years, 40-49 years, 50-59 years, and 60 years or older. Professional qualifications included midwife, registered nurse, licensed practical nurse, and advanced midwife, with multiple responses allowed. Job position was classified into four categories: staff nurse, charge nurse, head nurse, and other. Highest educational attainment was categorized as vocational school, junior college, four-year university, graduate school, or other.

Weekly working hours were categorized into five groups: less than 40 hours, 40-49 hours, 50-59 hours, 60-69 hours, and 70 hours or more. The number of full-time physicians was classified as fewer than 5, 5-9, 10 or more, or unknown. The number of full-time midwives was categorized as fewer than 10, 10-19, 20-29, 30 or more, or unknown. The number of advanced midwives was categorized as fewer than 5, 5-9, 10 or more, or unknown.

The annual number of deliveries was categorized into seven groups: none, fewer than 100, 100-199, 200-299, 300-399, 400 or more, or unknown. The ownership type of the employing institution was classified as public/publicly funded, national university hospital, private university hospital, or private hospital (excluding private university hospitals).

Regional characteristics were defined using Japan’s 344 secondary medical areas (SMAs), which were classified into three categories-large metropolitan areas, regional cities, and rural areas-based on a combination of population size and population density [20].

Control variables were selected based on previous nursing workforce and turnover intention studies reporting associations between workload, staffing structure, organizational support, and turnover intention among healthcare professionals [8,21]. In addition, organizational characteristics considered relevant to staffing structure and workload in obstetric departments were included.

Work seniority categories were determined based on categorizations commonly used in previous nursing workforce studies examining turnover intention and workforce retention [7,21], while also ensuring adequate sample sizes within each category for stable statistical analysis.

Statistical analysis

To identify factors associated with turnover intention-defined as intention to leave the current hospital or to leave the nursing or midwifery profession-multivariate logistic regression analyses were conducted. High-frequency turnover intention (defined as thinking about quitting at least once per week) was used as the dependent variable. Independent variables included respondents’ characteristics: age, professional qualifications, job position, highest educational attainment, weekly working hours, number of full-time physicians, number of full-time midwives, number of advanced midwives, annual number of deliveries, ownership type of the employing institution, and regional characteristics.

Turnover intention was operationalized using two self-reported items assessing the frequency of thoughts about leaving (1) the current hospital and (2) the nursing or midwifery profession. These two outcomes were assessed separately to distinguish intention to leave the current organization from intention to leave the nursing or midwifery profession. High-frequency turnover intention was defined as thoughts of leaving occurring at least once per week. Brief self-reported measures were adopted in the present nationwide survey to reduce respondent burden and improve feasibility.

The number of full-time physicians was included as an organizational characteristic reflecting the overall staffing structure and clinical workload environment of obstetrics departments. Physician availability may influence workflow distribution, emergency response burden, and interdisciplinary workload within obstetric care teams, and was therefore considered a potentially relevant contextual factor associated with turnover intention among nurses and midwives.

For all statistical analyses, p-values less than 0.05 were considered statistically significant. STATA version 17.0 (StataCorp LLC, College Station, USA) was used for all analyses. The final sample size was considered sufficient for logistic regression analyses based on the recommended events-per-variable approach for multivariate logistic regression models.

Ethical considerations

This study was approved by the Research Ethics Committee for Studies Involving Human Subjects at Tokyo Healthcare University (Approval No. 023-30B). The purpose of the study and measures to ensure secure data management were explained on the first page of the questionnaire. Participation was entirely voluntary. All analyses were conducted using anonymized data, and results were analyzed separately from personal information to ensure confidentiality.

## Results

Questionnaires were sent to 1,170 hospitals nationwide, and valid responses were obtained from 2,043 nurses and midwives at 474 hospitals, corresponding to a hospital-level response rate of 40.5%. The characteristics of respondents-including age, professional qualifications, job position, highest educational attainment, weekly working hours, number of full-time physicians, number of full-time midwives, number of advanced midwives, annual number of deliveries, ownership type of the employing institution, and regional characteristics-are summarized in Table 1.

Among the respondents, 1,640 were midwives, corresponding to 7.1% of the 23,109 hospital-employed midwives reported in the 2022 Survey of Medical Institutions conducted by the Ministry of Health, Labour and Welfare [18].

Regarding weekly working hours, the largest proportion of respondents worked 40-49 hours per week (1263, 61.8%), followed by those working 50 hours or more (244, 15.5%). Respondents working 60 hours or more per week accounted for 73(3.6%) of the total sample.

Figure 1 shows the frequency of turnover intention associated with working conditions among nurses and midwives working in obstetrics departments. Overall, 494 of 2,043 respondents (24.2%) reported thinking about leaving their current hospital at least once per week. Among the 895 respondents who answered the question regarding leaving the nursing or midwifery profession, 199 (22.2%) reported thinking about leaving the profession at least once per week.

**Figure 1:**
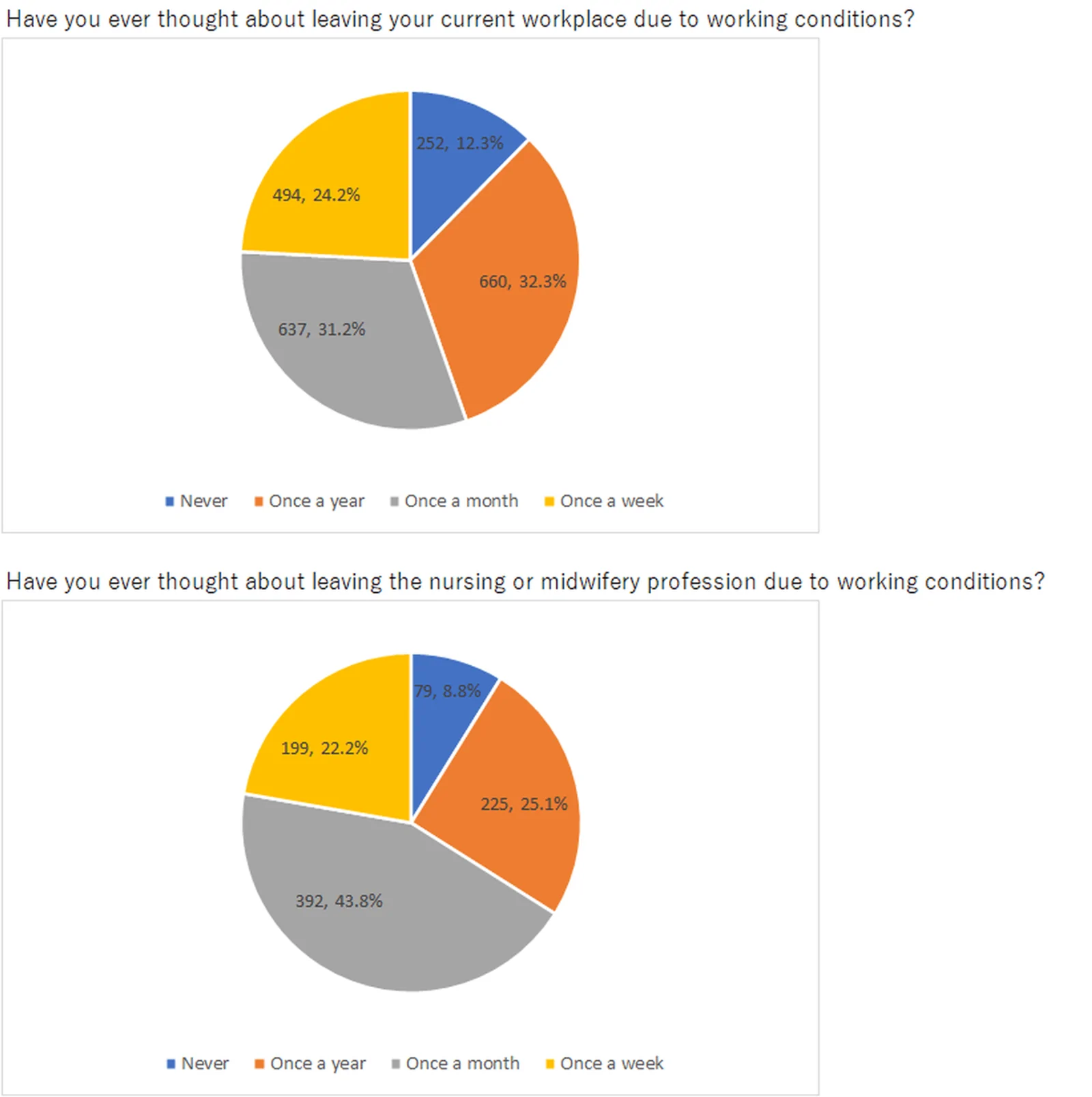
Frequency of turnover intention among nurses and midwives in obstetrics departments

These findings indicate that a substantial proportion of obstetrics nurses and midwives experience frequent turnover intention related to their working environment.

Table 2 presents the results of the multivariate logistic regression analyses examining factors associated with high-frequency turnover intention (defined as thinking about quitting at least once per week).

**Table 2:** Factors associated with intention to leave the current workplace (thinking about quitting at least once per week) OR: odds ratio, CI: confidence interval.

	OR	95% CI	p-value
Age			
<30	Reference		
30-39	1.05	0.77-1.43	0.76
40-49	0.82	0.59-1.14	0.23
50-59	0.84	0.58-1.22	0.36
≥60	0.56	0.26-1.23	0.15
Professional qualification			
Midwife	1.25	0.90-1.74	0.19
Registered nurse	1.10	0.86-1.42	0.44
Licensed practice nurse	0.75	0.43-1.29	0.29
Advanced midwife	0.99	0.73-1.34	0.95
Job position			
Staff nurse	Reference		
Charge nurse	0.78	0.57-1.07	0.13
Head nurse	0.47	0.29-0.75	0.00
Other	0.80	0.36-1.79	0.58
Highest educational attainment			
Vocational school	Reference		
Junior college	0.72	0.52-0.98	0.04
University	0.88	0.67-1.16	0.37
Graduate school	0.99	0.65-1.53	0.98
Other	0.43	0.21-0.87	0.02
Working Hours per Week			
<40 hours	Reference		
40~50 hours	1.13	0.86-1.47	0.38
50~60 hours	1.55	1.07-2.25	0.02
60~70 hours	2.16	1.13-4.11	0.02
≥70 hours	3.02	1.22-7.49	0.02
Number of full-time physicians			
<5	Reference		
5-9	1.01	0.77-1.32	0.94
≥10	1.20	0.78-1.85	0.40
Unknown	1.71	0.86-3.44	0.13
Number of full-time midwives			
<10	Reference		
10-19	0.62	0.45-0.87	0.01
20-29	0.65	0.43-0.98	0.04
≥30	0.66	0.40-1.09	0.10
Unknown	0.70	0.34-1.42	0.32
Number of advanced midwives			
<5	Reference		
5-9	1.07	0.81-1.41	0.62
≥10	0.58	0.37-0.91	0.02
Unknown	1.05	0.77-1.43	0.77
Annual number of deliveries			
None	Reference		
<200	0.90	0.43-1.87	0.77
200-400	1.24	0.58-2.65	0.59
≥400	1.55	0.71-3.40	0.28
Unknown	1.01	0.43-2.35	0.99
Foundational Entity of Employer			
Public	Reference		
Private	0.85	0.66-1.09	0.20
Public University	0.54	0.33-0.90	0.02
Private University	0.35	0.18-0.65	0.00
Employer’s Regional Classification			
Urban	Reference		
Intermediate	0.93	0.72-1.19	0.55
Rural	0.91	0.62-1.32	0.61

Intention to leave the current hospital

Table 2 presents the results for intention to leave the current hospital. Compared with respondents working fewer than 40 hours per week, those working 50-59 hours [odds ratio (OR) 1.55, 95% confidence interval (CI) 1.07-2.25, p = 0.02], 60-69 hours (OR 2.16, 95% CI 1.13-4.11, p = 0.02), and 70 hours or more (OR 3.02, 95% CI 1.22-7.49, p = 0.02) had significantly higher odds of turnover intention.

In contrast, holding a head nurse position was inversely associated with intention to leave the current hospital (OR 0.47, 95% CI 0.29-0.75, p < 0.001). Junior-college education (OR 0.72, 95% CI 0.52-0.98, p = 0.04) and other educational backgrounds (OR 0.43, 95% CI 0.21-0.87, p = 0.02) were also associated with lower odds compared with vocational-school education. Facilities with 10-19 midwives (OR 0.62, 95% CI 0.45-0.87, p = 0.01), 20-29 midwives (OR 0.65, 95% CI 0.43-0.98, p = 0.04), or at least 10 advanced midwives (OR 0.58, 95% CI 0.37-0.91, p = 0.02) had lower odds of turnover intention. Employment at public university hospitals (OR 0.54, 95% CI 0.33-0.90, p = 0.02) and private university hospitals (OR 0.35, 95% CI 0.18-0.65, p < 0.001) was also inversely associated with intention to leave the current hospital.

Intention to leave the nursing or midwifery profession

Table 3 presents the results for intention to leave the nursing or midwifery profession. Compared with respondents working fewer than 40 hours per week, only those working 70 hours or more had significantly higher odds of intending to leave the profession (OR 2.78, 95% CI 1.06-7.27, p = 0.04).

**Table 3:** Factors associated with intention to leave the nursing or midwifery profession (thinking about quitting at least once per week) OR: odds ratio, CI: confidence interval.

	OR	95% CI	p-value
Age			
<30	Reference		
30-39	1.30	0.93-1.82	0.13
40-49	1.00	0.70-1.43	1.00
50-59	1.08	0.72-1.61	0.71
≥60	0.71	0.30-1.69	0.44
Professional qualification			
Midwife	1.09	0.77-1.56	0.62
Registered nurse	1.12	0.86-1.48	0.40
Licensed practice nurse	0.66	0.36-1.22	0.18
Advanced midwife	0.89	0.64-1.25	0.51
Job position			
Staff nurse	Reference		
Charge nurse	0.82	0.59-1.14	0.24
Head nurse	0.38	0.22-0.67	0.00
Other	0.73	0.30-1.79	0.49
Highest educational attainment			
Vocational school	Reference		
Junior college	0.76	0.54-1.07	0.12
University	0.90	0.67-1.21	0.49
Graduate school	0.82	0.50-1.34	0.43
Other	0.62	0.31-1.26	0.19
Working Hours per Week			
<40 hours	Reference		
40~50 hours	1.14	0.85-1.52	0.37
50~60 hours	1.22	0.81-1.85	0.34
60~70 hours	1.57	0.77-3.24	0.22
≥70 hours	2.78	1.06-7.27	0.04
Number of full-time physicians			
<5	Reference		
5-9	0.88	0.65-1.17	0.38
≥10	1.16	0.73-1.84	0.54
Unknown	1.77	0.85-3.70	0.13
Number of full-time midwives			
<10	Reference		
10-19	0.70	0.49-1.01	0.06
20-29	0.74	0.47-1.15	0.18
≥30	0.69	0.40-1.10	0.19
Unknown	0.77	0.36-1.65	0.51
Number of advanced midwives			
<5	Reference		
5-9	1.05	0.78-1.42	0.75
≥10	0.57	0.35-0.94	0.03
Unknown	1.12	0.80-1.56	0.51
Annual number of deliveries			
None	Reference		
<200	0.80	0.37-1.74	0.58
200-400	1.16	0.53-2.58	0.71
≥400	1.40	0.62-3.20	0.42
Unknown	0.80	0.33-1.96	0.63
Foundational Entity of Employer			
Public	Reference		
Private	0.82	0.62-1.08	0.16
Public University	0.58	0.33-1.00	0.05
Private University	0.34	0.17-0.68	0.00
Employer’s Regional Classification			
Urban	Reference		
Intermediate	0.76	0.58-0.99	0.04
Rural	0.62	0.41-0.93	0.02

Conversely, holding a head nurse position was associated with lower odds of intending to leave the profession (OR 0.38, 95% CI 0.22-0.67, p < 0.001). Having at least 10 advanced midwives was also inversely associated with turnover intention (OR 0.57, 95% CI 0.35-0.94, p = 0.03). Employment at private university hospitals was associated with lower turnover intention than employment at public hospitals (OR 0.34, 95% CI 0.17-0.68, p < 0.001), while the association for public university hospitals was borderline (OR 0.58, 95% CI 0.33-1.00, p = 0.05). Compared with urban areas, intermediate areas (OR 0.76, 95% CI 0.58-0.99, p = 0.04) and rural areas (OR 0.62, 95% CI 0.41-0.93, p = 0.02) were associated with lower odds of intending to leave the profession.

## Discussion

This nationwide cross-sectional study examined turnover intention and its associated factors among nurses and midwives working in obstetrics departments in hospitals across Japan. The results showed that 494 of 2,043 respondents (24.2%) frequently considered leaving their current hospital, while 199 of 895 respondents (22.2%) who answered the corresponding question frequently considered leaving the nursing or midwifery profession altogether. Nurse turnover has repeatedly been shown to adversely affect the quality and safety of care [1,3,22], and securing and retaining nursing personnel remains a critical issue directly linked to the sustainability of healthcare systems as a whole [4].

In the present study, longer weekly working hours showed a clear dose-response association with intention to leave the current hospital, and working 70 hours or more per week was also associated with intention to leave the nursing or midwifery profession. Previous reviews and empirical studies have similarly demonstrated that long working hours and excessive workload are major determinants of turnover intention among nurses [5-8]. Within theoretical frameworks of turnover intention, working conditions and the work environment are regarded as central components in the formation of turnover intention [15], and the findings of this study are consistent with these theoretical perspectives.

Our findings are generally consistent with recent international studies reporting associations between workload, organizational support, burnout, and turnover intention among nurses and healthcare professionals [8,11]. To our knowledge, this is one of the first nationwide studies in Japan to examine turnover intention specifically among nurses and midwives working in obstetrics departments while focusing on organizational staffing structure and workforce support factors.

Extended working hours as well as night and shift work have been reported to increase turnover intention through heightened burnout and reduced job satisfaction [7,23]. Integrative reviews focusing specifically on turnover intention have also shown that psychological burden and decreased job satisfaction are closely associated with intention to leave [24]. Moreover, intervention studies and meta-analyses have consistently reported that long working hours are associated with deterioration in mental and physical health, thereby increasing the risk of workforce attrition [9,10].

This study further demonstrated that holding a head nurse position was associated with a lower level of turnover intention. Organizational research and studies in nursing management have shown that work environment quality and organizational support influence burnout and job retention [12-14], supporting the present findings.

Conversely, situations characterized by excessive workload and constraints on acting in accordance with professional values have been linked to the accumulation of moral distress [25], a psychological burden that may ultimately hinder long-term job retention.

The finding that facilities with a higher number of midwives and advanced midwives exhibited lower turnover intention is consistent with previous evidence indicating that nurse staffing levels and skill mix influence outcomes [26]. Studies focusing on midwives have reported that work environment characteristics and workload are associated with burnout [27], suggesting that adequate staffing may contribute to job retention through the alleviation of psychological burden.

Qualitative studies on midwives’ job retention have further demonstrated that mutual support among colleagues within the same profession and the quality of the work environment are key factors that enhance resilience in challenging situations [28]. The present finding that human resources within the same profession-specifically, the number of midwives and advanced midwives-were associated with lower turnover intention provides nationwide quantitative evidence that supports and extends these qualitative insights.

In Japan, chronic shortages of nursing staff persist, and harsh working conditions have been identified as a primary reason for turnover [18]. Obstetrics departments, in particular, are characterized by unpredictable and high-intensity workloads associated with deliveries and emergency care, and such structural features may contribute to psychological burden and influence job retention. Issues related to burnout and job retention among healthcare professionals, including midwives, represent an important concern internationally [29]. A previous systematic review and meta-analysis reported that burnout is prevalent among midwives and is associated with occupational and organizational factors [30]. The findings of this study offer insights that are relevant not only to Japan but also to global discussions on workforce retention in obstetric care.

These findings suggest that workforce retention in obstetric care settings should be addressed not only at the individual level but also through organizational and structural interventions. Strategies to reduce turnover intention may include improving staffing structures, strengthening managerial support, and enhancing professional support systems for midwives and nurses. These findings may also have relevance for other countries facing workforce shortages and increasing workload pressures in obstetric care settings.

From a practical perspective, reducing turnover intention in obstetric departments should not rely solely on individual resilience. Hospital administrators should consider maintaining adequate numbers of experienced midwives and advanced midwives, strengthening frontline nurse management, and monitoring excessive working hours. These organizational interventions may help improve workforce retention while maintaining the quality and safety of obstetric care.

Limitations

This study has several limitations. First, due to its cross-sectional design, causal relationships between working conditions and turnover intention cannot be established. Although long working hours and organizational factors may increase turnover intention, it is also possible that nurses and midwives who are considering leaving are more likely to be placed in, or perceive themselves to be in, more demanding working environments. Future longitudinal or interventional studies are needed to clarify causal pathways.

Second, because the present study used self-reported questionnaire data collected at a single time point, common method bias cannot be completely excluded. Harman’s single-factor test was not conducted, which should be considered a methodological limitation of the study.

Third, this survey was conducted using a web-based format, and participation was entirely voluntary, raising the possibility of selection bias. Nurses and midwives with stronger concerns about their working environment may have been more likely to respond. Nevertheless, the study surveyed 1,170 hospitals nationwide and obtained responses from 2,043 participants across 474 hospitals, providing a sample of considerable size and coverage for a study focusing on nurses and midwives working in obstetrics departments.

Fourth, turnover intention was assessed using two brief single-item questions rather than a validated multi-item psychometric scale. Although this approach reduced respondent burden and allowed us to distinguish between intention to leave the current hospital and intention to leave the nursing or midwifery profession, it may not fully capture the multidimensional nature of turnover intention and may be subject to measurement error. In addition, turnover intention was assessed based on the frequency of thoughts about leaving rather than actual turnover behavior. Although turnover intention does not necessarily result in actual turnover behavior, previous research and the Theory of Planned Behavior suggest that behavioral intentions are important predictors of subsequent behavior [16,17]. Therefore, turnover intention may still provide meaningful insight into workforce instability and retention risk among nurses and midwives. Future studies using validated multi-item measures and longitudinally observed turnover outcomes are warranted.

Fifth, this study was unable to fully account for other potentially important factors, such as salary level, detailed employment conditions, family circumstances, interpersonal relationships at work, and organizational culture. These unmeasured confounding factors may have influenced the observed associations between working conditions and turnover intention.

Finally, this study was conducted within the specific context of the Japanese healthcare system and obstetric care structure, and caution is required when generalizing the findings to other settings. However, the high workload characteristics of obstetrics departments and the roles of midwives share many similarities across countries, and the findings may provide useful insights for workforce retention strategies in obstetric care in other healthcare systems.

## Conclusions

This nationwide study demonstrated a dose-response association between longer working hours and intention to leave the current hospital, while working 70 hours or more per week was associated with intention to leave the nursing or midwifery profession. Organizational and contextual factors-including head-nurse roles, greater staffing of midwives and advanced midwives, university-hospital employment, and non-urban regional settings-were associated with lower turnover intention in one or both models. These findings suggest that workforce retention in obstetric care should be addressed not only at the individual level but also through organizational and structural interventions aimed at improving work environments and professional support systems.
